# A laser-induced mouse model of progressive retinal degeneration with central sparing displays features of parafoveal geographic atrophy

**DOI:** 10.1038/s41598-023-31392-3

**Published:** 2023-03-14

**Authors:** Adnan H. Khan, Sudha Priya Soundara Pandi, Jennifer A. Scott, Aida Sánchez-Bretaño, Savannah A. Lynn, J. Arjuna Ratnayaka, Jessica L. Teeling, Andrew J. Lotery

**Affiliations:** 1 Clinical and Experimental Sciences, Faculty of Medicine, University of Southampton, Southampton, UK; 2 Southampton Eye Unit, University Hospital Southampton NHS Foundation Trust, Southampton, UK; 3 Biological Sciences, Faculty of Environmental and Life Sciences, University of Southampton, Southampton, UK

**Keywords:** Experimental models of disease, Macular degeneration

## Abstract

There are no disease-modifying treatments available for geographic atrophy (GA), the advanced form of dry age-related macular degeneration. Current murine models fail to fully recapitulate the features of GA and thus hinder drug discovery. Here we describe a novel mouse model of retinal degeneration with hallmark features of GA. We used an 810 nm laser to create a retinal lesion with central sparing (RLCS), simulating parafoveal atrophy observed in patients with progressive GA. Laser-induced RLCS resulted in progressive GA-like pathology with the development of a confluent atrophic lesion. We demonstrate significant changes to the retinal structure and thickness in the central unaffected retina over a 24-week post-laser period, confirmed by longitudinal optical coherence tomography scans. We further show characteristic features of progressive GA, including a gradual reduction in the thickness of the central, unaffected retina and of total retinal thickness. Histological changes observed in the RLCS correspond to GA pathology, which includes the collapse of the outer nuclear layer, increased numbers of GFAP + , CD11b + and FcγRI + cells, and damage to cone and rod photoreceptors. We demonstrate a laser-induced mouse model of parafoveal GA progression, starting at 2 weeks post-laser and reaching confluence at 24 weeks post-laser. This 24-week time-frame in which GA pathology develops, provides an extended window of opportunity for proof-of-concept evaluation of drugs targeting GA. This time period is an added advantage compared to several existing models of geographic atrophy.

## Introduction

Age-related macular degeneration (AMD), a progressive disease that results in the loss of central vision, is predicted to affect 288 million people by 2040^1^. AMD has a multifactorial aetiology: the retina and choroid of the macular region are affected by photo-oxidation, inflammation and hypoxic stress^2–4^. This is in addition to underlying genetic factors and angiogenic stimuli^5–7^. Continuous exposure to these stresses affects the homeostasis of the chorioretinal tissues^8–11^, leading to the formation of drusen (clinically visible as yellow/white spots in fundus images) in the maculae of patients in the early stages of AMD.

Atrophic or ‘dry’ AMD leads to progressive degeneration of the retinal pigment epithelium (RPE) and choriocapillaris underlying the macula, with secondary photoreceptor damage and eventually, the clinical late-stage phenotype of geographic atrophy (GA). By contrast, neovascular, or ‘wet’ AMD (nAMD), is a result of choroidal neovascularisation, resulting in rapid vision loss^12^. The location and progression of GA determines visual prognosis. GA pathology proceeds with RPE and photoreceptor atrophy in the parafoveal region, sparing the fovea^13^. Hence, patients with dry AMD can retain substantial central vision for many years until pathology extends to the fovea^13^, a phenomenon known as foveal sparing. Histological evaluation of donor (post-mortem) eyes with GA exhibit not only the loss of photoreceptor outer segments and RPE, but also thinning of the outer nuclear layer (ONL) with choroidal atrophy and gliosis. The atrophy of these retinal layers result in the inner nuclear layer (INL) being proximal to Bruch’s membrane (BrM)^14–18^.

Although there is evidence that (AREDS) vitamin supplementation may reduce the risk of early AMD progressing further^19^, there are no curative treatments for the advanced forms of AMD. The application of available anti-vascular endothelial growth factor (VEGF) therapies serves to temporarily limit the damaging effects of nAMD. However, there is no such treatment for GA. The lack of a disease-modifying treatment for GA may be due to the complex nature of this phenotype. Animal models that exhibit key features of GA are crucial for testing potential new drugs. However, most models fail to fully recapitulate these GA features^20,21^. For example, the *Cryba1* conditional knockout mouse^22^, results in sub-retinal lesions and hypo/hyperpigmentation but fails to show BM thickening, ONL thinning or atrophic morphology. The Polyethylene glycol (PEG) model results in photoreceptor degeneration and hypopigmented RPE with drusen-like deposits but lacks features such as BM thickening and choroidal pathology^23^; The CCL2^−/−^/CX3CR1^GFP/GFP^ double-knockout mouse also shows photoreceptor degeneration and hypopigmented RPE with BM thickening, but lacks ONL thinning and characteristic wedge-shaped morphology at margins of GA lesions^24^. These models fail to provide convincing evidence of progressive atrophy observed in GA patients^21^.

We previously used an 810 nm laser to develop a mouse model of AMD that recapitulates the features of GA, including progressive outer retinal pathology^25^. In this study, we sought to refine this model by creating a retinal lesion with central sparing (RLCS), simulating parafoveal atrophy observed in patients with progressive GA. We monitored the lesion over 24 weeks and demonstrate features of progressive pathology, which could therefore act as a novel proof-of-concept tool for evaluating new drugs targeting GA.

## Results

### Laser-induced retinal lesions with central sparing (RLCS) produce progressive GA-like pathology over time with the development of confluent atrophic changes

**Figure 1:**
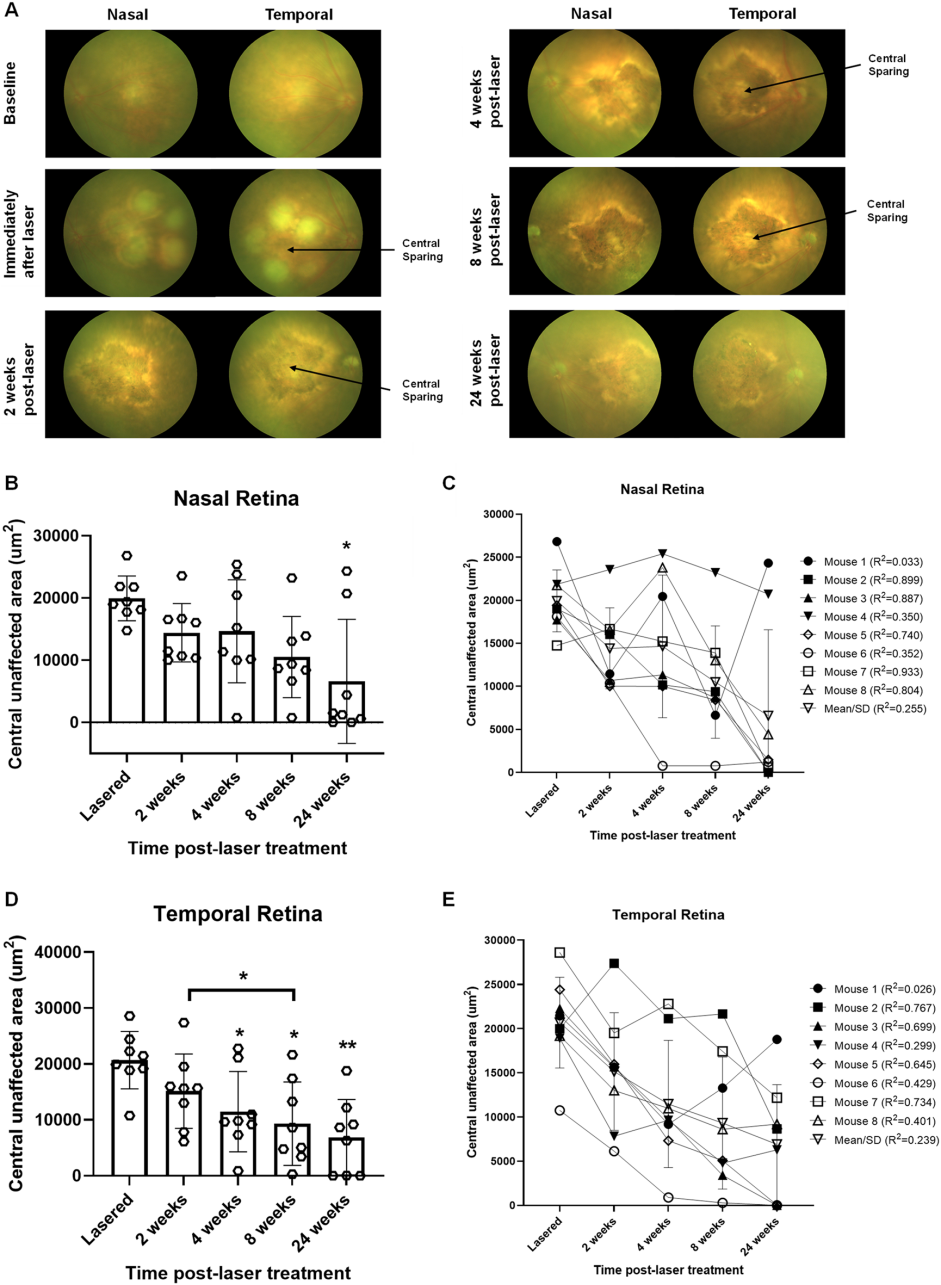
Laser-induced retinal lesions with central sparing (RLCS) progress to form GA-like atrophy with gradual reduction in area of the central untreated retina. (**A**) Representative colour fundus photographs are shown, taken at baseline (before laser treatment), immediately after laser treatment, and 2-, 4-, 8- and 24-weeks post laser treatment of C57BL/6 J female mice. Laser treatment consisted of creating a retinal lesion with 5 laser spots, applied circumferentially to encircle an area of central sparing. One eye of each mouse had a RLCS induced both nasal and temporal to the optic disc. Visible areas of central sparing in the lesions temporal to the optic disc are indicated with an arrow. The data shown in the graphs represent the central mean surface area that remained unaffected in the (**B**, **C**) nasal and (**D**, **E**) temporal RLCS at the indicated time points. Data are expressed as (**B**) and (**D**) mean ± SD (n = 8 eyes per group from 8 different mice) and comparison of the indicated time points made to the time of laser using one-way ANOVA testing and Bonferroni post-hoc testing; * *p* < 0.05, **p < 0.01. Data are also expressed (**C** and **E**) to demonstrate the dynamics of the central unaffected area in the individually treated eyes from the 8 different mice over the 24-weeks period, in addition to the mean ± SD of the group of eyes. A linear regression test was carried out for the individually treated eyes, with the *R*^*2*^ value shown for each mouse and for the group, to determine the relationship between central unaffected area and time point.

Colour fundus photographs (CFPs) were taken prior to laser treatment to rule out retinal pathology or media opacities (termed “baseline”) and various time points post-laser treatment (Fig. 1A). Laser treatment created a retinal lesion with 5 laser spots, applied circumferentially to encircle an area of central sparing, which we have termed a retinal lesion with central sparing (RLCS). A RLCS was induced nasal and temporal to the optic disc in one eye of each mouse. The fellow eye received no laser treatment and acted as an internal control. CFPs taken immediately following laser treatment showed bright yellow laser spots with peripheral appearances of swelling and a normal-appearing retina at the area of central sparing (Fig. 1A). At 2 weeks post-laser, an atrophic phenotype was observed with focal hyperpigmentation in the peripheral lasered area of the RLCS with well-demarcated yellow margins, and a normal-appearing retina in the central untreated area. With increasing time, there was an enlargement of the atrophic lasered area and encroachment into the central unaffected area, with complete closure of the central area by week 24 in 50% of the treated mice. Quantification of the central unaffected area showed a progressive reduction at 2, 4, 8 and 24 weeks post-laser treatment (Fig. 1B,C). On the lesion created on the nasal part of the retina (Fig. 1B), there was a progressive mean reduction in the central unaffected area (μm^2^) of 5512 μm^2^ (28%), 5284 μm^2^ (27%) and 9429 μm^2^ (48%) at weeks 2, 4, and 8 respectively, post-laser treatment. A significant reduction in the central unaffected area of the nasal lesion was observed at week 24 post-laser (mean reduction of 13,333 μm^2^ or 67%; *p* < 0.05). Linear regression testing was carried out to determine a relationship between the central unaffected area and time point post-laser for the individually-treated eyes and for the group as a whole (Fig. 1C). The *R*^2^ goodness of fit for each nasal lesion ranged from 0.033 to 0.933, and the overall *R*^2^ for the group was 0.255. A progressive and significant reduction in the central unaffected area (μm^2^) was observed in the lesion created in the temporal part of the retina (Fig. 1D) when measured at week 4 (mean reduction of 9213 μm^2^ or 45%; *p* < 0.05), week 8 (mean reduction of 11,369 μm^2^ or 55%; *p* < 0.05) and week 24 (mean reduction of 13,787 μm^2^ or 67%; *p* < 0.01) when compared to the time of laser treatment. The effect in the temporal lesion was most pronounced at the 24-week time point post-laser. On linear regression testing, the *R*^*2*^ goodness of fit for each temporal lesion for the individually treated eyes ranged from 0.026 to 0.767, and the overall *R*^2^ for the group was 0.239 (Fig. 1E). The overall findings indicate a reduction of the central unaffected area from week 2, which continues to progress up to week 24 in both the nasal and temporal lesion.

### Longitudinal OCT scans of RLCS demonstrates features characteristic of GA progression

**Figure 2:**
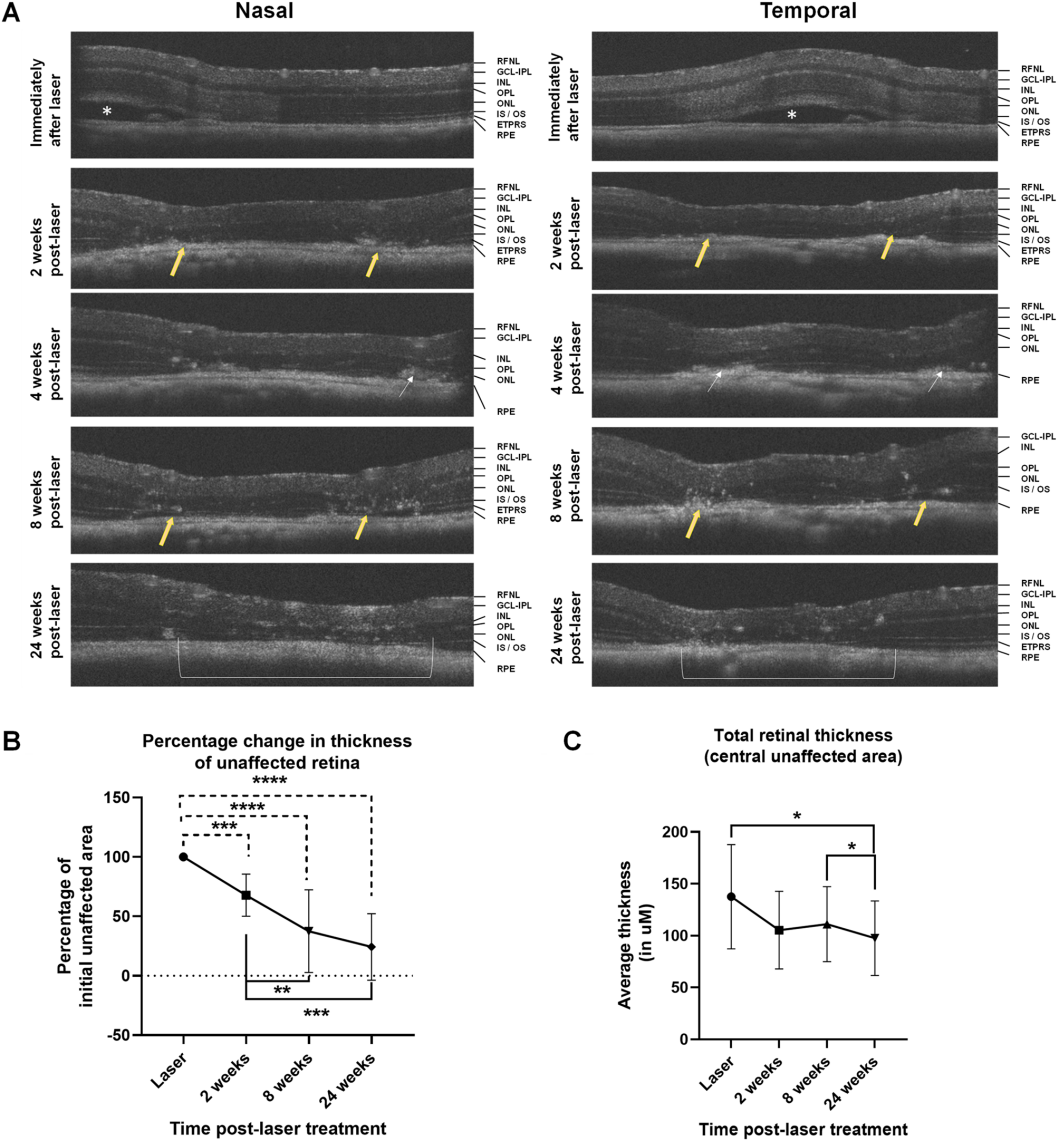
Laser-induced RLCS demonstrate optical coherence tomography (OCT) features characteristic of parafoveal GA progression. (**A**) Representative OCT scans from mouse eyes taken immediately after laser exposure, and at 2-, 4-, 8- and 24-weeks post laser treatment of C57/BL6J female mice to create a RLCS both nasal and temporal to the optic disc. Sub-retinal fluid accumulation immediately following laser treatment is shown (white asterisk), in addition to focal absence of the IS/OS junction at the laser spots (yellow arrow), ONL thinning, and development of hyperreflective areas (white arrow) and progression/merging of two atrophic spots to form a single lesion (white bracket) in lasered eyes. The data shown in the graphs represent the (**B**) mean percentage change in thickness of the central untreated retina, i.e. the percentage that remained unaffected and (**C**) total retinal thickness at the indicated time points post-laser treatment. Data are expressed as mean ± SD (n = 8 eyes per group from 8 different mice) and comparison of the indicated time points made to the time of laser using non-parametric Wilcoxon signed-rank testing.* *p* < 0.05, **p < 0.01, ***p < 0.001, ***p < 0.0001. Retinal nerve fibre layer (RNFL), ganglion cell layer—inner plexiform layer (GCL-IPL), inner nuclear layer (INL), outer plexiform layer (OPL), outer nuclear layer (ONL), photoreceptor inner segment / outer segment junction (IS/OS), photoreceptor end tips (ETPRS) and retinal pigment epithelium (RPE).

Optical coherence tomography (OCT) scans taken immediately after laser treatment demonstrated an acute response, with subretinal fluid accumulation at the site of the laser/RLCS in both the nasal and temporal lesions (Fig. 2A). After 2 weeks, outer retinal/structural changes were observed on OCT scans, in the vicinity of the lasered areas. These structural changes included the presence of hyper-reflective outer retinal pathology, with loss of the photoreceptor inner segments (IS) and outer segments (OS), and collapse of the inner nuclear layer (INL) and outer nuclear layer (ONL), which suggest photoreceptor degeneration. At the 24-week time point post-laser treatment, OCT scans demonstrated progressive closure of the central unaffected area, adjacent to the lasered lesions and regression of hyper-reflectivity from 4 weeks post-laser onwards. Of note, by 24 weeks post-laser, OCT images revealed a confluent atrophic lesion in 50% of treated mice, similar to results obtained by CFP. Quantification shows a significant and progressive decrease in size of the unaffected area at week 2 (% of baseline; *p* < 0.001), week 8 (% of baseline; *p* < 0.0001) and week 24 (% of baseline *p* < 0.0001) when compared to baseline (Fig. 2B). This was consistent with data from CFP measurements. In comparison to the week 2 time point post-laser treatment, there was a significant reduction of the percentage unaffected area at both the week 8 (*p* < 0.01) and week 24 (*p* < 0.001) timepoints, confirming a progressive reduction of the central unaffected retina over time. A significant reduction in the total retinal and individual retinal layer thickness was observed at the week 24 (*p* < 0.05) time point, relative to when treated with the laser (Fig. 2C). Interestingly, no significant differences were observed at the 2-week time point, suggesting that overall structural changes to the retina are progressive, rather than acute or transient (Fig. 2C).

### Histological changes observed in RLCS correspond to GA pathology

**Figure 3:**
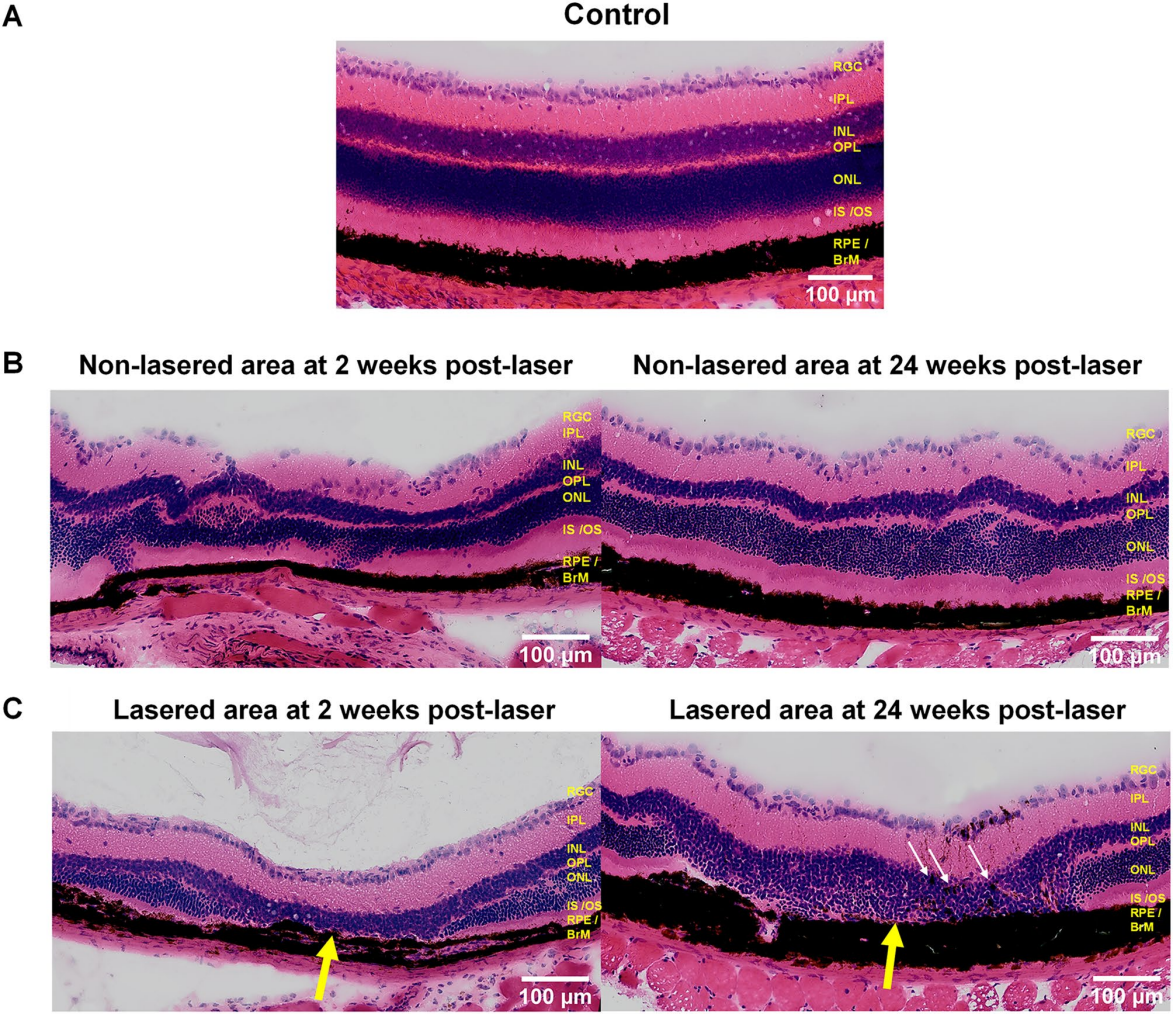
Histological changes observed in RLCS are typical of GA pathology. Representative Haematoxylin and Eosin (H&E) stained retinal sections from (**A**) naïve control mice; (**B**) non-lasered areas of retina and (**C**) corresponding laser spot areas at 2 weeks (left) and 24 weeks (right) after laser treatment to induce a RLCS. Sections taken from lasered eyes demonstrate loss of the IS/OS junction, with apparent migration of RPE into the INL (white arrows), intact BM and choroid and collapse of the INL and ONL into the lesion site (yellow arrows). Retinal ganglion cell layer (RGC); inner plexiform layer (IPL), inner nuclear layer (INL), outer plexiform layer (OPL), outer nuclear layer (ONL), photoreceptor inner segment / outer segment junction (IS/OS), retinal pigment epithelium—Bruch’s membrane interface (RPE/BrM).

Next, we examined H&E-stained chorioretinal sections for evidence of retinal pathology following laser treatment compared to naïve control mice (Fig. 3A). Tissue sections from areas peripheral to the lasered region (non-lasered, Fig. 3B) were compared to sections containing the laser-induced lesion (lasered, Fig. 3C). Areas of laser-induced lesions show the presence of an intact RPE-Bruch’s membrane complex (Fig. 3C), suggesting that the outer blood-retinal barrier remained intact post-laser, similar to tissues from control mice. At both week 2 and week 24 time points post-laser treatment, changes to the RPE were demonstrated, including evidence of hyperplastic RPE and migration of pigment into the INL, particularly at 24 weeks. Furthermore, retinal damage was observed, including absence of IS/OS, changes to the ONL and collapse of both the INL and ONL in lasered areas. Histological evaluation therefore confirmed photoreceptor degeneration and RPE changes at the sites of laser-induced retinal lesions. Adjacent (non-lasered) areas of retina displayed normal architecture at both week 2 and week 24 timepoints post-laser treatment (Fig. 3B).

### Inflammatory changes observed in RLCS have features of early GA pathology

**Figure 4:**
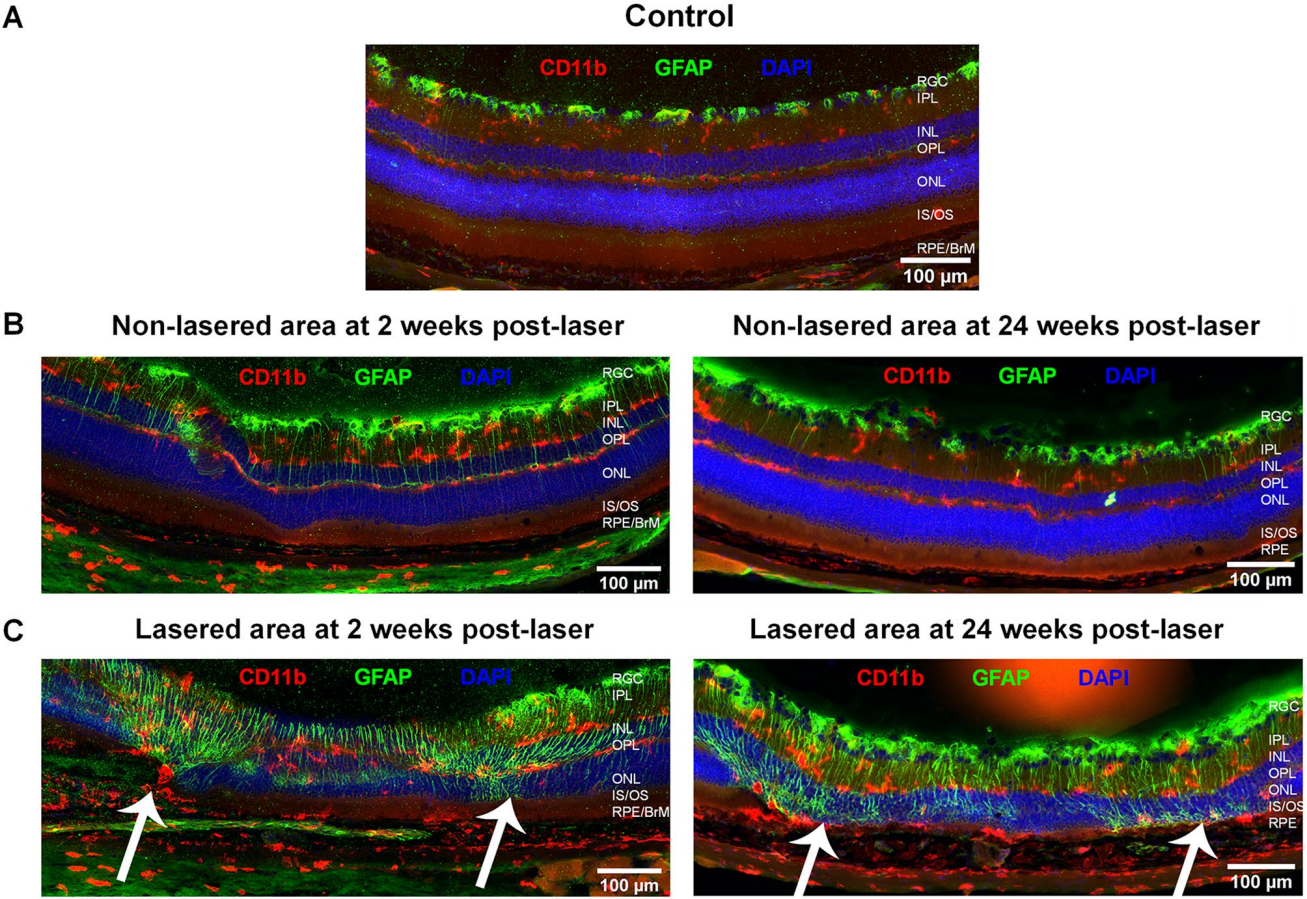
Increased GFAP-positive cell protrusions from RGCs into the site of RLCS in addition to increased CD11b + cells. Representative immunofluorescence images of stained retinal sections from (**A**) control mice; (**B**) non-lasered areas of retina and (**C**) corresponding laser spot areas at 2 weeks (left) and 24 weeks (right) after laser treatment to induce a RLCS. Retinal sections were probed with anti-GFAP (green), expressed by Müller cells and astrocytes, and anti-CD11b (red), expressed by microglial and/or other myeloid cells. Nuclei were stained with DAPI (blue). Chorioretinal sections show evidence of increased GFAP-positive protrusions extending from the RGCs to the site of the lesion, as well as increased CD11b + cells at the site of the laser lesions (white arrows) at 2 and 24 weeks post-laser treatment. Retinal ganglion cell layer (RGC); inner plexiform layer (IPL), inner nuclear layer (INL), outer plexiform layer (OPL), outer nuclear layer (ONL), photoreceptor inner segment/outer segment junction (IS/OS), retinal pigment epithelium—Bruch’s membrane interface (RPE/BrM).

**Figure 5:**
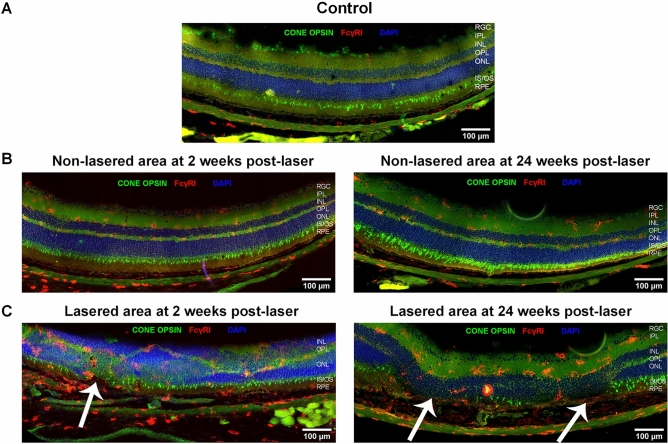
Increased presence of FCγRI + cells and cone photoreceptor damage after induction of RLCS. Representative immunofluorescence images of stained retinal sections from (**A**) naïve control mice; (**B**) non-lasered areas of retina and (**C**) corresponding laser spot areas at 2 weeks (left) and 24 weeks (right) after laser treatment to induce a RLCS. Retinal sections were probed with antibodies against cone opsin (green), anti-FcγRI (red) and DAPI nuclear stain (blue). Tissues show evidence of the absence of cone photoreceptors and increased FCγRI + cells at the lesion sites (white arrows). Retinal ganglion cell layer (RGC); inner plexiform layer (IPL), inner nuclear layer (INL), outer plexiform layer (OPL), outer nuclear layer (ONL), photoreceptor inner segment/outer segment junction (IS/OS), retinal pigment epithelium—Bruch’s membrane interface (RPE/BrM).

**Figure 6:**
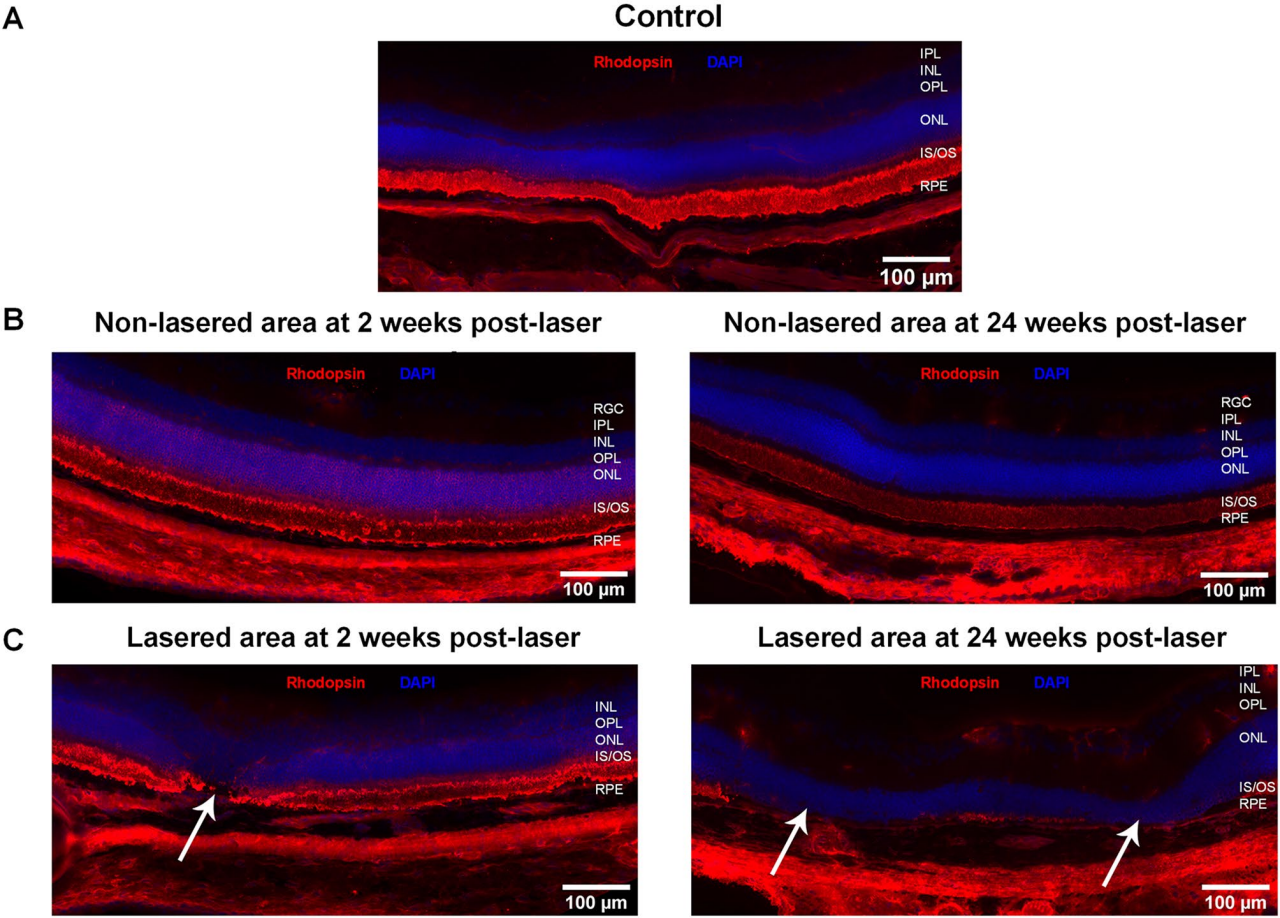
Rod photoreceptor damage after induction of RLCS. Representative immunofluorescence images of chorioretinal tissues from (**A**) control mice; (**B**) non-lasered areas of retina and (**C**) corresponding laser spot areas at 2 weeks (left) and 24 weeks (right) after laser treatment to induce a RLCS. Retinal sections were probed with anti-rhodopsin (red) and stained with DAPI (blue). Tissues show evidence of the absence of rod photoreceptors at the laser lesion sites (white arrows). Rod/photoreceptors remained unaffected in non-lasered areas. Retinal ganglion cell layer (RGC); inner plexiform layer (IPL), inner nuclear layer (INL), outer plexiform layer (OPL), outer nuclear layer (ONL), photoreceptor inner segment/outer segment junction (IS/OS), retinal pigment epithelium—Bruch’s membrane interface (RPE/BrM).

Immunofluorescence staining was undertaken in chorioretinal tissues from retina at 2 weeks and 24 weeks post-laser treatment. In naïve, control retinal tissue, glial fibrillary acidic protein (GFAP) was detected only in the retinal ganglion cell (RGC) layer (Fig. 4A). Non-lasered areas of retina from mice that had undergone laser treatment demonstrated GFAP expression in the RGC layer, similar to non-lasered mice (Fig. 4B). At 2 weeks post-laser treatment, increased expression of GFAP was observed in the inner nuclear layer (INL) (Fig. 4B), which resolved at 24 weeks. In contrast, lasered areas of retinae demonstrated increased GFAP expression and cell processes extending towards the RPE at both week 2 and week 24 time points post-laser (Fig. 4C). This confirms activation of Müller glial cells and/or astrocytes at areas of laser treatment. Resident CD11b + cells and FcγRI + cells were restricted to the IPL and OPL regions in retinae from naïve, control mice (Figs. 4A and 5A), and in adjacent non-lasered areas of the week 2 and week 24 post-laser treated retinae (Figs. 4B and 5B). In contrast, increased numbers of CD11b + and FcγRI + cells were observed in the lasered areas at the week 2 and week 24 time points post-laser treatment, and in particular around the RPE (Figs. 4C and 5C). Photoreceptor degeneration in lasered areas was confirmed by loss of cone opsin expression (Fig. 5C) and loss of rhodopsin expression (Fig. 6C) at both week 2 and week 24 time points compared to non-lasered mice (Figs. 5A and 6A). Expression of rhodopsin and cone opsin immunoreactivity in adjacent non-lasered areas of the retina (Figs. 5B and 6B) are similar to retinae from naïve, control mice, suggesting that the retinal pathology in the lasered areas didn’t affect adjacent/untreated area. Rods, rather than cone photoreceptors dominate the mouse retina, hence there is significantly less variable cone positivity in the retinal sections. The findings indicate increased inflammatory activity in response to, or as a result of, photoreceptor and RPE degeneration in the lasered eyes, which subsequently included the central unaffected/non-lasered region.

## Discussion

In this study, we developed a laser-induced mouse model of GA, with progressive retinal pathology including foveal sparing-like GA features. Fundus images of the laser-treated retina demonstrate that the atrophic pathology includes an intact RPE-BrM. The laser-induced lesions, which initially surround an area of unscathed retina akin to the spared foveal region in GA patients^26^, merge over time to form a confluent area of focal atrophy, recapitulating the progressive loss of this important region. OCT scans confirm findings from CFP by showing a similar percentage change in the central non-lasered/unaffected area. Histology and immunofluorescence analyses of chorioretinal tissues demonstrates increased inflammation, loss of cone opsin and rhodopsin expression levels, and RPE abnormalities with unaltered BrM, consistent with histopathological findings in donor/post-mortem GA tissues. Furthermore, the loss of cone opsin and rhodopsin expression levels (suggestive of photoreceptor degeneration), ultimately includes the central non-lasered region. Interestingly, GFAP activation initially observed at 2 weeks post-laser, appeared to spread from the lasered regions to the central (laser-spared) region by week 24. Therefore, a progressive inflammatory response is observed from week 2 onwards, which remains active until week 24 post-laser, suggesting features of progressive retinal inflammatory changes. However, this migration of immune cells did not reach statistical significance.

Despite current ongoing trials (e.g. the complement C3 inhibitor Pegcetacoplan^27^), there are no disease-modifying treatments available for GA. As a result, there is an ongoing demand for animal models which recapitulate salient features of GA. Compared to other species, rodent models of GA are the most common as they are generally easy to handle, breed and genetically modify^21^. For example, the short lifespan of mice means that disease phenotypes can be studied within relatively short timeframes. Current acute injury models of GA include sub-retinal injection of compounds such as sodium iodate^28^ and polyethylene glycol^23^. However, these models have the added risk of operative complications such as retinal tears and/or detachment during the procedure. Variable amounts of sub-retinal and vitreous haemorrhage also contribute to an additional inflammatory stimulus. In addition, there is variability of lesion size caused by the volume of the compound ultimately being delivered into the right location (in the subretinal space). By contrast, advantages of the laser model described in this study are its non-invasive nature (non-invasive to the eye), enabling the generation of highly reproducible lesions that are consistent in size as demonstrated by CFP and OCT readouts. Hence, an advantage of this model is that robust and rapid readouts could be obtained following treatment with new drugs, by measuring structural changes in retinal thickness on OCT scans. Most genetically-modified mouse models of GA demonstrate pathology similar to those induced after sub-retinal injections described above^21^. This includes POS degeneration, a thickened BrM, hypopigmented and vacuolated RPE and choroidal thinning^21^. This is in contrast to the laser model described here, where progressive GA pathology over the course of 24 weeks is demonstrated, including RPE degeneration, POS/IS loss, ONL thinning, inflammation and well-demarcated wedge-shaped atrophy similar to the acute injury rat model^29^.

The ageing human retina is characterised by para-inflammation, which has been described as maintaining tissue homeostasis, and has features between basal and inflammatory states, resulting in low-grade inflammatory-based clearance of noxious stimuli^30^. This inflammation is heightened in AMD with chronic inflammatory responses occurring due to the recruitment and activation of resident microglial cells and/or peripheral myeloid cells to the site of injury^3,31^. The laser-induced mouse model described here demonstrated increased numbers of activated microglial cells, associated with increased GFAP expression, to the lasered area of atrophy, which persisted until the 24-week timepoint. More importantly, GFAP activation and protrusions of Müller glial cells from the ganglion layer to the lasered regions at the 2-week time point, eventually extends to the central unaffected/non-lasered area by 24 weeks, suggesting the progressive inflammatory responses akin to those reported in GA eyes^16^.

There are several limitations to the use of a laser-induced model to study GA, prominent of which is that it fails to account for the chronic destabilising and pro-inflammatory conditions in the outer retina typical of GA pathology. However, our findings reported herein demonstrate the likely recruitment of Müller glial cells and FcγRI + inflammatory cells to the site of laser treatment, which gradually extends to encompass the central unaffected/non-lasered region akin to the spared fovea. Our previous study using an 810 nm laser to develop a single confluent GA-like lesion in the central mouse retina showed evidence of complement and inflammasome activation in lasered regions^25^. Another limitation of this study is the use of mice (which do not possess an anatomical fovea) to model human foveal sparing. It has been suggested that the phenomenon of foveal sparing could be due to fovea-specific factors^32^. Despite this, the progression of retinal degeneration in the 810 nm laser-induced mouse model to involve the central unaffected area provides some parallel to the rate of loss of foveal sparing area in humans, demonstrated in a clinical study to be relatively similar on comparison of several retinal dystrophies^32^. In the past, development of the acute laser-induced neovascular AMD mouse model, although imperfect, led to initial validation of anti-VEGF inhibitors^20^. Similarly, in the 810 nm laser mouse model presented here, the ultimate loss of cone opsin and rhodopsin expression levels in the central non-lasered region, similar to that observed during the progression of parafoveal GA to involve the fovea, might provide validation of drugs currently under development, such as the complement C3 inhibitors^27^. Lastly, it could be possible that subthreshold laser damage to the central unaffected area, not initially visible on CFP or OCT imaging in this study, could contribute to the progression of retinal degeneration ultimately seen by 24 weeks. Studies at the ultrastructural level may also provide further details of outer retinal pathology, and in particular any changes to the unaffected/non-lasered region mimicking the spared foveal region in GA.

In summary, this model presents a further refinement of the laser-induced GA model which we described earlier, but one that incorporates a highly sought-after feature of progressive GA pathology; foveal sparing. As such, this model presents an altogether new tool and the first of its kind, to study the fate of functional retinal tissues surrounded by gradual, encroaching atrophic retinal degeneration. It is relatively easy to incorporate into a laboratory merely requiring wild type mice and the relevant laser and OCT equipment. Exploitation of this model has the potential to provide new insights into the biological mechanisms underlying GA pathology. Additionally, the time frame of progressive atrophy in this model provides a convenient opportunity for proof-of-concept efficacy evaluation of drugs targeting GA.

## Methods

### Animal experiments

All experiments were approved by the local Animal Welfare and Ethical Review Body (University of Southampton). All experiments were performed in accordance with relevant guidelines and regulations, including the ARVO statement for the use of Animals in Ophthalmic and Vision Research. Reporting of animal experiments follows recommendations in the ARRIVE guidelines. All experiments in this study were performed on three-month old C57BL/6 J female mice (n = 22, including control mice). The mice were maintained in a standard 12 h dark/12 h light cycle, with food and water available ad libitum. Mice were administered reversible anaesthesia with a combination of ketamine (80 mg/kg, Chanelle) and xylazine (8 mg/kg, Bayer) in 0.1 mL of saline. Mouse pupils were maximally dilated using 1% tropicamide and 2.5% phenylephrine hydrochloride eye drops (Bausch & Lomb). The corneas of mice were kept hydrated throughout laser treatment and imaging procedures carried out with repeated topical application of artificial tears (Viscotears, Alcone). Anaesthesia was reversed at the conclusion of the procedure with 10% antisedan in sterile saline at 8 μl/g of mouse weight. For terminal procedures, mice were anesthetised with 0.1 ml pentobarbitol and trans-cardially perfused with 0.9% NaCl (Sigma-Aldrich, UK) containing 5 units/ml heparin sulfate (CP Pharmaceuticals, UK).

### Laser treatment to induce atrophic retinal lesions with central sparing (RLCS)

Laser was applied to one eye of anaesthetised mice (n = 16) with dilated pupils using a fibre-coupled 810 nm continuous wave diode laser (Carlton, Leicester, UK) with a laser injector lens connected to a Micron III Retinal Imaging Microscope (Pheonix Research Labs, Pleasanton, CA, USA). The laser injector has a camera for obtaining colour fundus photographs, enabling orientation and visualisation of the lasered area. The 810 nm laser has a near-infrared wavelength enabling deeper penetration of retinal layers and is mainly absorbed by the RPE-choroid^33^. The retina was targeted with a focused laser beam (400 μm diameter) at a power of 1000 mW for 500 ms per spot. This results in circa 100 mW power output at the retina. A retinal lesion was induced with 5 laser spots, applied circumferentially to encircle an area of central sparing, termed retinal lesion with central sparing (RLCS). The laser spots of the RLCS were applied in close apposition to enable the lesions to develop confluence. The fundus of each mouse was oriented such that an RLCS was induced both nasal and temporal to the optic disc. Six mice were used as naïve, non-lasered controls and were not lasered. Effects of laser treatment to the retina were evaluated longitudinally by colour fundus photographs (CFP) and optical coherence tomography (OCT) at 2, 4, 8 and 24 weeks post-laser. Eyes were enucleated at 2 and 24 weeks post-laser for histology and immunofluorescence analysis.

### In vivo* imaging*

Colour fundus photographs (CFP) were acquired with a camera and bright field imaging (450–650 nm) incorporated into the laser injector and visualised using the Phoenix Micron III Retinal Imaging Microscope Software. Progressive development/closure of the RLCS was measured using FIJI software (www.fiji.sc). Two investigators undertook data analysis, and one was blinded (JS) to control and experimental groups. The interquartile ratio was used to represent the variability between analyses.

OCT imaging was undertaken using the Leica Envisu R2200 VHR SDOIS Mouse Imaging System (Leica, IL, USA) with mice positioned as previously described^34^. We obtained 1.4 mm^3^ volumetric scans (consisting of 100 B-scans and 1000 A-scans per B-scan) using InVivoVue 2.4 Diver software (Leica Microsystems, IL, USA) with the mouse positioned such that the optic nerve head appeared in one corner of the image. Colour fundus photographs were mapped to an overlying 11 × 11 grid that incorporated as much of the RLCS as possible. OCT images were manually segmented as previously described^34^. Retinal layers for each point on the grid were selected, and the thickness of the neurosensory retina and its eight constituent layers were calculated from the manual segmentation data points. Progression of laser-induced retinal lesions was evaluated by the percentage affected area and thickness from the central (laser-spared) area at all-time points. The eight constituent retinal layers (manually segmented and thickness calculated as previously described^34^) are as follows: Retinal nerve fibre layer (RNFL), ganglion cell layer—inner plexiform layer (GCL-IPL), inner nuclear layer (INL), outer plexiform layer (OPL), outer nuclear layer (ONL), photoreceptor inner segment/outer segment junction (IS/OS), photoreceptor end tips (ETPRS) and retinal pigment epithelium (RPE).

### Immunohistochemistry and immunofluorescence

Following trans-cardial perfusion, eyes were harvested 2 and 24 weeks post laser treatment and were embedded in optimal cutting temperature medium followed by rapid freezing with isopentane on dry ice. Immunohistochemistry with haematoxylin/eosin (H&E) staining was carried out on 16 µm thick cryo-sectioned eyes as previously described^35^. For immunofluorescence experiments, chorioretinal sections were dried, fixed in 100% ice-cold ethanol and washed in 1 × PBS prior to blocking in 10% normal goat serum. The sections were incubated overnight using the following primary antibodies: rabbit anti-human GFAP (Dako, Denmark), rat anti-mouse CD11b (5C6; Thermo Fisher, UK), rat anti-mouse FcγRI (Clone 152–9, Biorad, CA, USA), mouse anti-rhodopsin (Merck, New Jersey, USA), rabbit anti-opsin (Merck, New Jersey, USA) at 4 °C. The following day, the sections were washed and incubated with the following secondary antibodies: goat anti-rabbit IgG-AF488, goat anti-rat IgG (H + L)-AF568 and goat anti-mouse IgG1 AF568 (Thermo Fisher, UK) for 1 h at room temperature. Nuclei were counterstained with DAPI and mounted with mowiol mounting medium (Sigma-Aldrich, UK). Images were obtained using an Olympus VS110 virtual slide scanning microscope with 20 × objective using extended focus imaging settings. All images were exported to FIJI software (www.fiji.sc) to add scale bars using microscope calibration setting parameters. Immunofluorescent staining was undertaken in the total retinal region of the lasered areas for GFAP, CD11b, FCγRI, DAPI, rhodopsin and cone opsin staining.

### Statistical analysis

Data obtained from imaging (fundoscopy and OCT) and was assessed for normality of variance, one-way ANOVA and Bonferroni post-hoc testing, in addition to linear regression analysis, using Graphpad Prism v8 (CA, USA). OCT data obtained from laser-induced retinal lesions temporal and nasal to the optic disc were comparable and thus combined for analysis. Non-parametric Wilcoxon signed-rank testing was used to analyse the percentage of retina (not exposed to laser treatment) affected over time, including the thickness of the retina and constituent layers over time. A value of *p* < 0.05 was deemed to be statistically significant.

## Data Availability

The datasets generated and analysed during the current study are available from the corresponding authors on reasonable request.
